# Supplementary material for: Causes and consequences of sex-chromosome turnovers in Diptera
Source: Evol Lett. 2026 Mar 12;10(3):253–68. doi: 10.1093/evlett/qrag003 (PMC13231274; doi:10.1093/evlett/qrag003)
Supplement: qrag003_Supplemental_Files [file qrag003_supplemental_files.zip › Supplementary_Datasets.zip!/Supplementary_Datasets/Dataset_2_Graphical_representation_outgroups/Culicidae_Anopheles_merus_chromosomes.pdf]

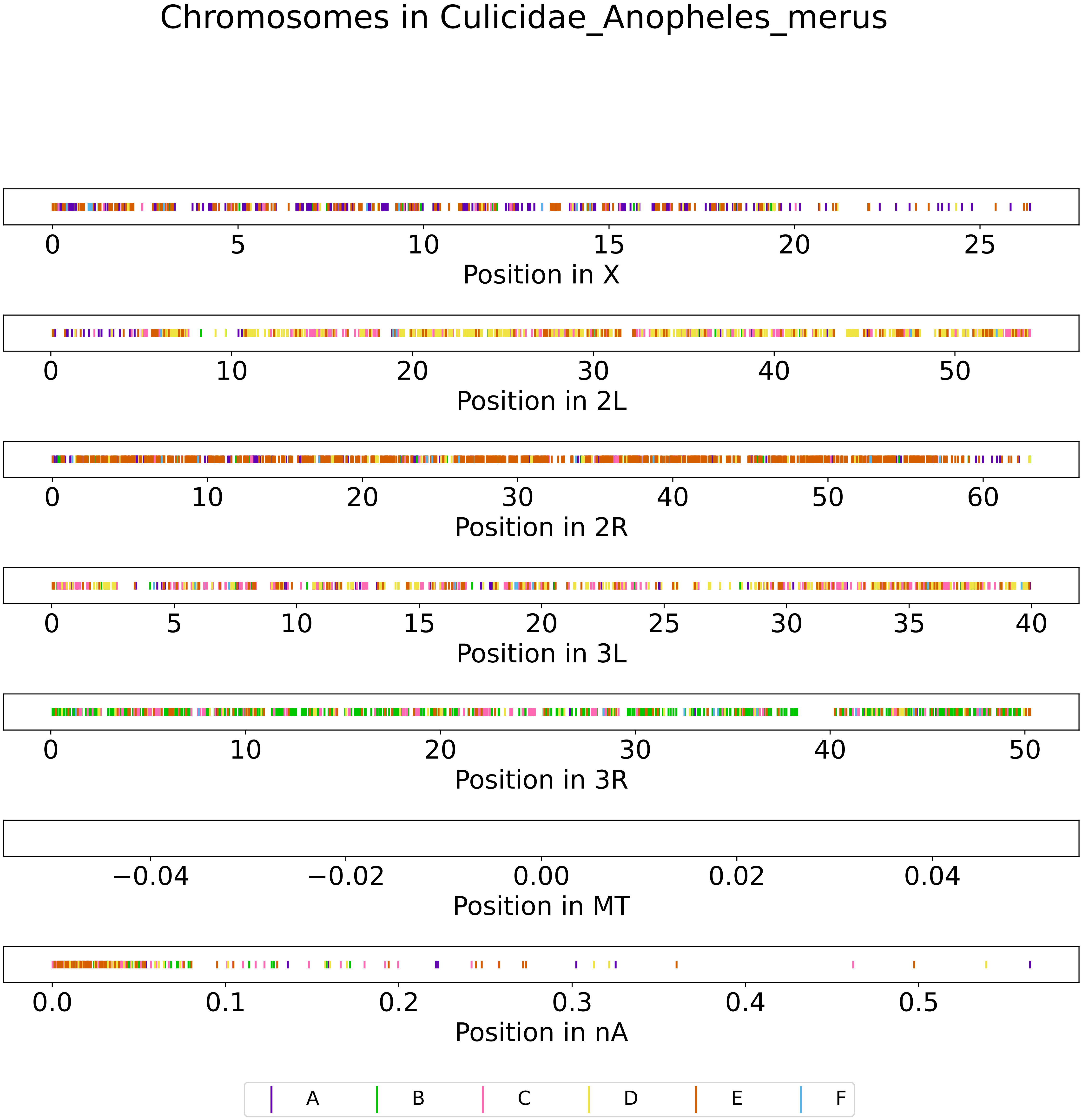
Chromosomes in Culicidae_Anopheles_merus
0
5
10
15
20
25
Position in X
0
10
20
30
40
50
Position in 2L
0
10
20
30
40
50
60
Position in 2R
0
5
10
15
20
25
30
35
40
Position in 3L
0
10
20
30
40
50
Position in 3R
−0.04
−0.02
0.00
0.02
0.04
Position in MT
0.0
0.1
0.2
0.3
0.4
0.5
Position in nA
A
B
C
D
E
F
